# The Late Sir B. W. Richardson

**Published:** 1896-11-28

**Authors:** 


### The Late Sir B. W. Richardson.

We regret to have to record the death of Sir Benjamin W. Richardson, which occurred on November 21st as the result of an attack of apoplexy. In him we lose a frequent and welcome contributor to the columns of The Hospital, one of the most active-minded men in the medical profession, and one who will be missed by the public as much as by his colleagues.

Always a bright and versatile intelligence, always an interesting talker, always able to introduce new thoughts into old subjects, his position in the public eye, and especially in the eye of that large and increasing number of the public who look on the use of alcohol with disfavour, was fully maintained to the last as a trusted physician, a sympathetic adviser, and a philosopher who was always both ready and able to explain, to simplify, and to make clear problems in medical science which had seemed complicated and obscure.

With many in his profession it is probable that, of recent years, he had to some extent dropped out of touch. Ready as he always was to infuse originality into the topics which he studied, he was by no means so ready to accept the stream of new ideas which other writers have during the last twenty years poured into the general fund; and interesting—sparkling, indeed—as was much of his writing, one cannot but see in it a certain apartness, and, during recent years at any rate, a tendency to appeal to his own experience and his own experiments, whether of yesterday or of years ago, to the exclusion of much which medical men of to-day are wont to accept as proven. His position on the alcohol question also, no doubt, had its influence, and although whenever he spoke at the medical societies his remarks were listened to with interest, and with the respect due to a careful observer and an original investigator, it can hardly be said that of late he had that hold upon his professional brethren as an elucidator and a path-finder which he had when, years before, they presented him with a splendid microscope and a purse of a thousand guineas as a token of their appreciation of the character and extent of his labours for the improvement of scientific medicine.

Sir Benjamin Richardson was born at Somerby, in the county of Leicester, in 1828, and took the degree of M.D. at the University of St. Andrews in 1854. In the early years of his professional life he practised at Barnes, at first as assistant to, and afterwards as partner with, the late Dr. Willis. At that time cholera was prevalent in England, and he concerned himself much with its investigation. He busied himself also with the various topics which came up for discussion at the several medical societies. But the ties of general practice were not to his mind, although it may be said that in his care and personal interest, and in his memory of the personal traits and family peculiarities of his patients, he was a general practitioner to the last; and he soon took up his residence in London with the object of devoting himself the more completely to the medical and physiological researches in which he took, and to the time of his death continued to take, such interest. In 1856 he obtained the Astley Cooper triennial prize of £300 for the best essay on the coagulation of the blood, and this was followed soon after by the publication of a paper on the deposit of fibrin in the heart. The result of this work was a considerable amount of consulting practice, and, much as he was doing in many other fields, it is probable that to his work on coagulation and on cardiac thrombosis he owed much of his success as a practitioner.

His work, however, was unending and incessant. Writing, lecturing, practising as a physician, investigating both as a chemist and as a physiologist, inventing as he went along instruments for artificial respiration, substances for producing anæsthesia, styptic colloid for the healing of wounds and the arrest of bleeding, local anæsthesia for small operations, and finally a lethal chamber for the painless destruction of superfluous animals, he, nevertheless, in the midst of all his work, was not without poetic fancy and imagination, and twenty years ago he showed by his paper on "Hygeia, or a City of Health," how the chemist and the physiologist could also be a dreamer of dreams.

But the dream was vivid and was well told, and although probably few of us could now describe exactly what it was all about, it took the public taste, and exercised an influence in the direction of sanitary righteousness greater than many a far more serious contribution to science.

Sir Benjamin Richardson lived a life of constant interest and of ceaseless activity, and, although he died all too young, better so perhaps than that a mind so agile should have lived on to watch its own decay—the cruellest penalty of a too long life.

